# From caregiving burden to income transformation: intergenerational employment effects of long-term care insurance

**DOI:** 10.3389/fpubh.2025.1606448

**Published:** 2025-06-10

**Authors:** Zhiying Li, Longhua Zheng, Hong Xu

**Affiliations:** ^1^School of Government, University of Chinese Academy of Social Sciences, Beijing, China; ^2^English Department, Jining Shiqiao Middle School, Jining, China; ^3^School of Public Administration and Policy, Shandong University of Finance and Economics, Jinan, China

**Keywords:** long-term care insurance, older household income, employment promotion, income inequality, Difference-in-Differences

## Abstract

**Background:**

Long-term care insurance (LTCI) has been introduced in China to address the challenges posed by an aging population. This study examines its impact on the income of older adult households, using data from the China Health and Retirement Longitudinal Study (CHARLS) from 2011 to 2020. The analysis further explores heterogeneity and implications for income inequality.

**Methods:**

A Difference-in-Differences (DID) approach is employed, with 12 pilot cities as the treatment group and non-pilot cities as the control group. The analysis is based on 33,435 valid observations of individuals aged 60 and above. The main outcome variable is per capita household income, which has been deflated to 2011 constant prices using the Consumer Price Index (CPI). Demographic and regional characteristics are controlled for robustness.

**Results:**

LTCI significantly increases the income of older adult households, particularly in multi-child families and among younger older adult (under 75). Its impact is weaker in low- or no-child households and for those aged 75 and above. Additionally, LTCI contributes to reducing income inequality by enhancing income distribution among low-income older adult households.

**Discussion:**

The findings highlight the role of LTCI in improving economic security and narrowing income disparities among older adult households. To enhance its effectiveness, policy adjustments should account for family structure and age composition. These insights provide empirical support for optimizing LTCI to foster a more inclusive social security system.

## 1 Introduction

Over the past four decades, China’s rapid economic growth has significantly increased household income levels. However, its “prosper the rich first” strategy has contributed to widening income disparities. Since 2003, China’s Gini coefficient has remained above 0.46, surpassing the internationally recognized warning line of 0.4 (1), highlighting severe income inequality. As the country shifts its development model from high-speed growth to high-quality development, optimizing income distribution and addressing socio-economic imbalances have become pressing challenges (2). Simultaneously, China’s rapidly aging population further exacerbates income inequality. The proportion of people aged 65 and above increased from 9.4% in 2001 to 15.6% in 2024, with the total number reaching 220 million (3). Alongside aging, the number of disabled older adults has risen to over 35 million as of 2024 and is projected to grow further (4).

In smaller family structures, this surge in care needs places substantial economic burdens on households, affecting labor supply and intergenerational support, thereby impacting household income. The rising costs of caregiving crowd out household consumption and investment, while family members may reduce labor participation or withdraw from the workforce entirely, leading to income losses. This phenomenon, described as “one disabled person, whole family unbalanced,” imposes financial strain and uncertainty on older households.

To address these challenges, China introduced a pilot Long-Term Care Insurance (LTCI) program in 2016 to alleviate caregiving burdens, reduce family care costs, and improve access to care services. Since its initial pilot in 15 cities, LTCI has undergone two major development phases, gradually expanding from local trials to national implementation, covering 49 cities by 2020. Existing research extensively explores LTCI’s effects on older adults’ health and caregiving burdens. Studies have confirmed that LTCI reduces medical expenditures (5–7), improves self-care ability (8), cognitive function (9), and general health outcomes (10), and lowers mortality rates (11). However, urban–rural disparities persist, with LTCI decreasing medical costs in rural areas but increasing them in urban regions (12), highlighting the critical role of resource allocation in shaping policy outcomes.

In terms of economic outcomes, social security policies have been shown to affect household income and inequality. While social security has improved income levels for low-income older households and reduced rural poverty (13, 14), its effect on income inequality remains contested. Some studies indicate that social security policies narrow income gaps (15, 16), while others argue they may intensify wealth concentration and income polarization (17, 18), suggesting that the impact of social security on inequality is context-dependent and warrants further analysis.

Despite substantial literature on LTCI’s health benefits and its alleviation of caregiving burdens, significant gaps remain. First, most research focuses on LTCI’s impact on medical expenditures, overlooking its potential to influence family income through enhanced labor market participation among both older adults and their children. By reducing caregiving burdens, LTCI may improve labor supply, potentially increasing household income. Second, while literature discusses LTCI’s health and caregiving effects, few studies address its implications for income inequality. Third, current research largely neglects the temporal dimension of LTCI’s impact, lacking insights into its short-term versus long-term effects and potential diminishing or lagging outcomes.

This study aims to bridge these gaps by analyzing the impact of LTCI on household income and income inequality. Using panel data from the China Health and Retirement Longitudinal Study (CHARLS) from 2011 to 2020, the Difference-in-Differences (DID) method is applied to empirically examine how LTCI influences household income through intergenerational employment effects. Additionally, this study explores LTCI’s potential impact on income inequality and conducts heterogeneity analysis to reveal variations across socio-economic backgrounds, providing targeted empirical evidence for optimizing long-term care policies and advancing the goal of common prosperity.

## 2 Theoretical mechanism

As a risk-sharing mechanism, LTCI plays a crucial role in stabilizing the economy within the context of an aging society. LTCI provides systematic long-term care coverage, thus alleviating the financial pressure faced by older adults and their families due to care needs. The government or social insurance institutions bear part of the long-term care costs through the LTCI system, enabling family members to reduce the direct financial expenditures caused by caregiving responsibilities (19). This mechanism not only reduces financial uncertainty for families but also increases disposable income and, to some extent, enhances income stability.

From the perspective of the precautionary saving theory, income uncertainty is an important factor driving household saving behavior (20, 21). In the absence of insurance coverage, households tend to increase precautionary savings to cope with potential economic shocks. However, excessive precautionary savings may lead to an imbalance in household resource allocation, thereby crowding out funds that would otherwise be used for productive investment and consumption, suppressing economic participation, and further limiting income growth (22–24). LTCI reduces the uncertainty of future care expenditures, thereby enhancing the risk-bearing capacity of insured households, lowering the demand for excessive savings, and improving investment willingness and financial asset allocation optimization (25). This mechanism not only helps in the effective use of family resources but also promotes broader economic participation, thus broadening income sources and achieving long-term income growth. Based on this, the following hypothesis is proposed:

*H1:* LTCI promotes income growth in households with older adults.

Individual labor supply is influenced by a combination of health status, time constraints, and economic incentives (26). In older adults, deterioration in health, increased long-term caregiving responsibilities, and limitations in pension systems often lead to a reduction in labor supply, or even early exit from the labor market (27). Health capital theory (28) emphasizes the decisive role of health in determining an individual’s labor capacity and employment behavior. LTCI, by providing professional care services and economic support, reduces the decline in labor capacity due to health issues or caregiving responsibilities, and minimizes work interruptions or early exit due to health problems (29). Studies have shown a positive relationship between improved health and labor market participation, especially among older workers, where improved health can extend working years, enhance labor productivity, and increase household income (30). Furthermore, LTCI can reduce uncertainty about future health expenditures, thus reducing the demand for excessive precautionary savings, improving consumption willingness, and enhancing labor supply motivation for older adults (31). Based on this, the following hypothesis is proposed:

*H2a:* LTCI promotes income growth in households with older adults by improving older adults’ employment.

Individuals need to weigh the opportunity costs of different activities when making economic decisions (32). The distribution of economic resources and labor supply decisions within a household are interdependent, and the redistribution of caregiving responsibilities may affect the employment status of the children of older adults (33). In traditional family models, households often bear the primary caregiving responsibility for older adults, which leads to a reduction in children’s work hours or forces them to exit the labor market (34). Long-term career development depends on continuous work experience and skill accumulation, and if children are forced to reduce labor participation or interrupt their career development due to caregiving responsibilities, their long-term income potential will be impacted (35). LTCI, by providing professional care, reduces children’s caregiving time, enabling them to increase working hours or enter higher-paying jobs, thus improving household income. In some regions, LTCI pilot programs also provide cash subsidies or care allowances, allowing households to arrange caregiving resources more flexibly, further optimizing labor allocation and improving overall economic efficiency (36). Based on this, the following hypothesis is proposed:

*H2b:* LTCI promotes income growth in households with older adults by improving children’s employment.

In the absence of formal caregiving coverage, low-income households with older adults often struggle to afford high caregiving costs, leading to a reduction in disposable income and exacerbating economic vulnerability (37). Poor households with limited resources lack sufficient financial buffers to cope with unexpected health shocks, making them more susceptible to falling into poverty traps (38). According to social insurance theory (39), social insurance systems reduce economic fluctuations caused by health events through risk-sharing mechanisms, enhancing income stability. LTCI, through fund collection and cost-sharing, provides more equal access to care resources for low-income older adults, thereby reducing income inequality caused by caregiving costs. Studies on countries such as the Netherlands, Germany, and the United States have shown that social insurance systems can buffer income shocks, reduce income volatility, and thereby decrease income inequality (40, 41).

Liquidity constraint theory suggests that limited liquidity hinders income accumulation in low-income households. High caregiving costs may force households to use savings or sell assets, undermining their long-term income growth capacity (42). LTCI, by providing financial support, reduces the caregiving burden on families, preventing them from falling into financial difficulties due to high caregiving expenses, thereby enhancing their ability to accumulate income and improving the sustainability of future income growth. Research has shown that inclusive social insurance systems can effectively narrow income disparities (43). For example, the implementation of agricultural insurance has improved agricultural total factor productivity, promoted agricultural income growth, and narrowed the income gap between urban and rural areas (44). These findings further validate the positive role of insurance systems in optimizing income distribution. Based on this, the following hypothesis is proposed:

*H3:* LTCI reduces income inequality in households with older adults.

**Figure 1:**
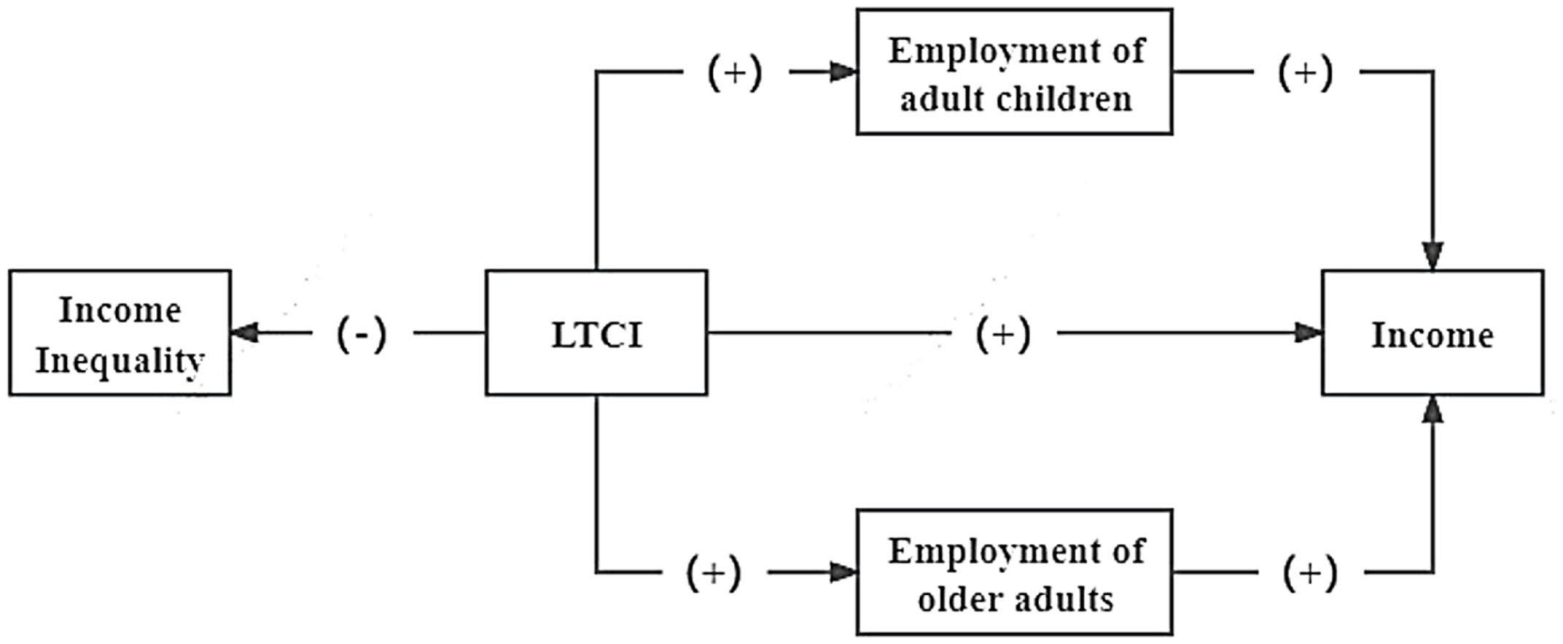
Research framework.

In summary, the theoretical framework for this study is illustrated in Figure 1.

## 3 Methods

### 3.1 Data

The data for this study is sourced from the China Health and Retirement Longitudinal Study (CHARLS) database. This survey, organized by Peking University, covers 150 districts and counties across the country and collects comprehensive information on individuals’ socioeconomic and health status, making it highly relevant for the analytical needs of this study. Additionally, the cross-sectional design of CHARLS from 2011 to 2020 enables this study to employ the DID method to assess the causal effects of the LTCI pilot policy. This approach effectively controls for unobserved factors related to time variation and individual differences, enhancing the robustness of the identification strategy. To ensure analytical precision, this study selects data from the years 2011, 2013, 2015, 2018, and 2020, and restricts the sample to individuals aged 60 and above and their families. After excluding missing values, a final sample of 33,435 valid observations is obtained.

### 3.2 Variable

The core explanatory variable in this study is a binary indicator of whether city c implemented the LTCI pilot program in year t. The study selects the 15 cities in the initial batch of pilots as the treatment group. However, since the CHARLS database does not include data for respondents from Nantong, Changchun, and Shihezi, the treatment group is limited. Therefore, the 12 pilot cities, excluding these three, are defined as the experimental group, while all non-pilot cities covered by CHARLS serve as the control group. If individual i resides in a city c that has implemented the pilot program in year t, the indicator is assigned a value of 1; otherwise, it is assigned a value of 0.

The dependent variable is the income of the senior household. To control for the effect of household size on income, per capita household income is calculated based on survey data to more accurately measure the household’s economic situation. Given that taking the natural logarithm of income may result in negative values or sample loss, as per the study by Liu and Ma (45), this study uses the raw income values directly for analysis. Additionally, to account for fluctuations in price levels, household income is deflated using the Consumer Price Index (CPI) with 2011 as the base year, ensuring comparability of data across different periods. Furthermore, to minimize the influence of extreme values on the estimates, a 1% two-tailed winsorization is applied to the household income variable, and observations with missing values are excluded to improve the robustness and representativeness of the data.

Other factors that may affect senior household income are also controlled, including individual characteristics of the senior (residence, gender, age, education level, ethnicity, pension insurance, medical insurance, and chronic illness status) and family characteristics (marital status and household size). Time fixed effects and city fixed effects are also included to control for macroeconomic factors and regional differences that may influence the results. Descriptive statistics for the variables are presented in Table 1.

**Table 1:** Variable definitions and descriptive statistics.

Variable type	Variable name	Variable definition and assignment	Mean	SD	Min	Max
Dependent Variable	income	*Per Capita* Household Income Adjusted for CPI (in Ten Thousand Yuan)	1.04	1.424	0	23.09
Core explanatory variable	did	Implementation of the LTCI pilot × year	0.045	0.206	0	1
Control variables	residence	Respondent’s residence (Urban = 0, Rural = 1)	0.584	0.493	0	1
	gender	Respondent’s gender (Female = 0, Male = 1)	0.493	0.5	0	1
	age	Respondent’s actual age at the time of the survey	68.905	6.953	60	120
	education	Respondent’s education (Below Primary = 1, Primary = 2, Secondary = 3, High School and Above = 4)	1.81	1.007	1	4
	ethnicity	Ethnicity of Respondents (Non-Han = 0, Han = 1)	0.934	0.248	0	1
	pension	Respondent’s pension insurance status (No pension insurance = 0, Any pension insurance = 1)	0.845	0.362	0	1
	health insurance	Respondent’s health insurance status (No health insurance = 0, Any health insurance = 1)	0.949	0.221	0	1
	chronic disease	Whether the respondent has chronic diseases (No = 0, Yes = 1)	0.827	0.378	0	1
	marital status	Respondent’s marital status (Not married = 0, Married = 1)	0.807	0.394	0	1
	household size	Number of family members of the respondent	2.86	1.613	1	16

### 3.3 Model specification

In the field of economic and social research, the DID method has been widely applied to policy effect evaluations. The basic idea is to treat policy pilot programs as a form of exogenous “quasi-natural experiment.” The implementation of LTCI policies may generate pre- and post-differences in the pilot areas, or create differences between pilot and non-pilot areas at the same point in time. By utilizing these two types of differences in regression analysis, the net effect of LTCI policies on household income can be effectively identified. Therefore, this study treats the LTCI pilot policy as a quasi-natural experiment and applies the DID method to assess its implementation effects. The baseline regression model is specified as follows:


(1) $$\text{Incom}e_{\text{ict}}=\alpha_{0}+\beta_{1}\text{LTC}I_{\text{ct}}+\beta_{2}\text{Control}s_{\text{ict}}+\gamma_{c}+\delta_{t}+\varepsilon_{\text{ict}}$$

Where: Subscripts *i*, *c*, and *t* represent individual, city, and time, respectively. $\text{Incom}e_{\text{ict}}$ is the dependent variable, indicating the income level of household *i* in city *c* in year *t*. $\text{LTC}I_{\text{ct}}$ is the core independent variable, representing whether city *c* implemented the LTCI pilot in year *t*, and it represents the interaction term between the treatment group city dummy variable and the pre- and post-pilot LTCI policy dummy variable. $\text{Control}s_{\text{ict}}$ represents individual and household-level control variables. $\gamma_{c}$ and $\delta_{t}$ are city fixed effects and time fixed effects, respectively. $\varepsilon_{\text{ict}}$ is the random error term. Robust standard errors are used, with clustering at the individual level.

The validity of the estimation in Equation 1 relies on the assumption of parallel trends, meaning that in the absence of policy intervention, the trends in the dependent variable for both the treatment and control groups should be parallel. To test this assumption, this study adopts the event study approach from Beck et al. (46) and examines the dynamic effects of the pilot policy. The dynamic effect analysis model is shown in Equation 2:


(2) $$\text{Incom}e_{\text{ict}}=\alpha_{0}+\sum_{t=2011}^{2020}\beta_{t}\text{LTC}I_{\text{ct}}+\beta_{2}\text{Control}s_{\text{ict}}+\gamma_{c}+\delta_{t}+\varepsilon_{\text{ict}}$$

Where $\beta_{t}$ represents the estimated values corresponding to the years 2011–2020. The definitions of other variables remain the same as in Equation 1.

To address potential bias in the DID estimation arising from other policy interventions, this study employs a triple difference-in-differences (DDD) approach to more accurately estimate the net effect of the LTCI policy. Building on the DID framework, this study introduces control and treatment groups that were not affected by the LTCI policy. By comparing the effects of the policy impact group with the control group, and subtracting the effects from groups that were not influenced by the policy, the net effect of LTCI can be derived. This approach effectively controls for heterogeneity across cities and individuals, enhancing the precision of the estimates. The DDD model is shown in Equation 3:


(3) $$\text{Incom}e_{\text{ict}}=\alpha_{0}+\beta_{1}\text{DD}D_{\text{ct}}+\beta_{2}\text{Control}s_{\text{ict}}+\gamma_{c}+\delta_{t}+\varepsilon_{\text{ict}}$$

The third interaction term (DDD: Treat × Post × Insurance) is used to capture the heterogeneity of policy implementation conditions. The Insurance variable indicates whether the individual meets the eligibility criteria for LTCI. For example, in some regions (e.g., Chengde and Qiqihar), the target group only includes individuals covered by employee health insurance, while in other regions (e.g., Changchun and Qingdao), it includes both employees covered by employee health insurance and urban residents covered by urban residents’ health insurance. In still other areas (e.g., Shanghai and Jingmen), both employee health insurance and rural–urban residents’ health insurance beneficiaries are included. Given the differences in insurance eligibility requirements across regions, this study constructs a triple difference interaction term based on regional eligibility conditions to accurately identify the policy responses of different insured groups. This approach helps avoid systematic bias in traditional DID methods, providing policy effect estimates with greater external validity.

In the study of income inequality, macro-level group inequality is typically measured by indicators such as the concentration index (47) and the Gini coefficient (48), which are suitable for assessing overall income inequality at the national or regional level. In contrast, micro-level individual inequality focuses more on income disparities within groups, and the Kakwani relative deprivation index (49) effectively quantifies income differences between individuals, making it particularly suitable for analyzing income inequality at the household level. Based on the theory of relative deprivation, individuals with lower incomes experience greater deprivation and higher levels of income inequality. This study draws on the work of Fukushige et al. (50) and Turguttopbaş (51), using the Kakwani relative deprivation index to measure income inequality among households. Let Y represent the reference group, with a sample size of n, and the total income distribution vector is sorted as y = ($y_{1},y_{2},y_{3},y_{n-1},y_{n}$), where $y_{1}\leq y_{2}\leq y_{3}\leq y_{n-1}\leq y_{n}$. Therefore, compared to the *j*-th individual, the relative deprivation index $\text{RD}(y_{j},y_{i})$ of the *i*-th individual is expressed as:


(4) $$\text{RD}(y_{j},y_{i})=\{\begin{array}{c} y_{j}-y_{i}\;\text{if}\;y_{j}>y_{i}\\ \;0\;\text{if}\;y_{j}\leq y_{i} \end{array}$$

Based on Equation 4, the average income relative deprivation index $\text{RD}(y,y_{i})$ for the *i*-th individual is shown in Equation 5:


(5) $$\text{RD}(y,y_{i})=\frac{1}{n\mu_{Y}}(n_{y_{i}}^{+}\times \mu_{y_{i}}^{+}+n_{y_{i}}^{+}\times y_{i})$$

Where $\mu_{y}$ is the mean income of all samples in the reference group Y, $n_{y_{i}}^{+}$ is the number of older individuals in the reference group Y whose income exceeds $y_{i}$, and $\mu_{y_{i}}^{+}$ is the mean income of older individuals in the reference group Y whose income exceeds $y_{i}$.

## 4 Results

### 4.1 Baseline regression

Table 2 presents the baseline regression results for the impact of the LTCI pilot policy on the income of older households. Model 1 includes only the core explanatory variables, and the interaction term coefficient is positive and significant. Models 2 and 3 further add individual characteristics of the older population and household characteristics, with the interaction term coefficient remaining significant, indicating robust results. Model 4 incorporates time and regional fixed effects, with the interaction term coefficient of 0.1556 being significant at the 1% level, suggesting that the LTCI pilot policy effectively increased the income level of older households, supporting Hypothesis 1.

**Table 2:** Baseline regression results.

Variable	Model 1	Model 2	Model 3	Model 4
did	0.5415^***^	0.4094^***^	0.3779^***^	0.1556^***^
	(0.0327)	(0.0307)	(0.0305)	(0.0476)
gender		−0.1570^***^	−0.1612^***^	−0.1037^***^
		(0.0150)	(0.0151)	(0.0183)
residence		−0.8611^***^	−0.8589^***^	−0.7529^***^
		(0.0156)	(0.0155)	(0.0269)
age		−0.0016	−0.0033^***^	−0.0079^***^
		(0.0010)	(0.0011)	(0.0014)
education		0.4284^***^	0.4193^***^	0.3267^***^
		(0.0076)	(0.0075)	(0.0110)
ethnicity		−0.1075^***^	−0.1325^***^	−0.1667^***^
		(0.0280)	(0.0279)	(0.0478)
pension		0.3375^***^	0.3215^***^	0.2126^***^
		(0.0179)	(0.0178)	(0.0175)
health insurance		0.1162^***^	0.1208^***^	0.1156^***^
		(0.0372)	(0.0370)	(0.0297)
chronic disease		0.0271	0.0161	0.0122
		(0.0186)	(0.0185)	(0.0199)
marital status			0.1334^***^	0.0862^***^
			(0.0204)	(0.0237)
household size			−0.0900^***^	−0.0558^***^
			(0.0046)	(0.0042)
_cons	1.0494^***^	0.7003^***^	1.0238^***^	1.4863^***^
	(0.0074)	(0.0837)	(0.0919)	(0.1170)
time-fixed effects	No	No	No	Yes
regional-fixed effects	No	No	No	Yes
*N*	42,163	33,436	33,435	33,435
adj. *R*^2^	0.0064	0.2356	0.2446	0.3464

Standard errors in parentheses.

^*^p < 0.1, ^**^p < 0.05, ^***^p < 0.01.

### 4.2 Robustness checks

To ensure the robustness of the research findings, multiple methods were employed for robustness checks, including parallel trend tests, placebo tests, propensity score matching difference-in-differences (PSM-DID), exclusion of key pilot provinces, triple difference (DDD), and alternative dependent variables.

#### 4.2.1 Parallel trend test

**Figure 2:**
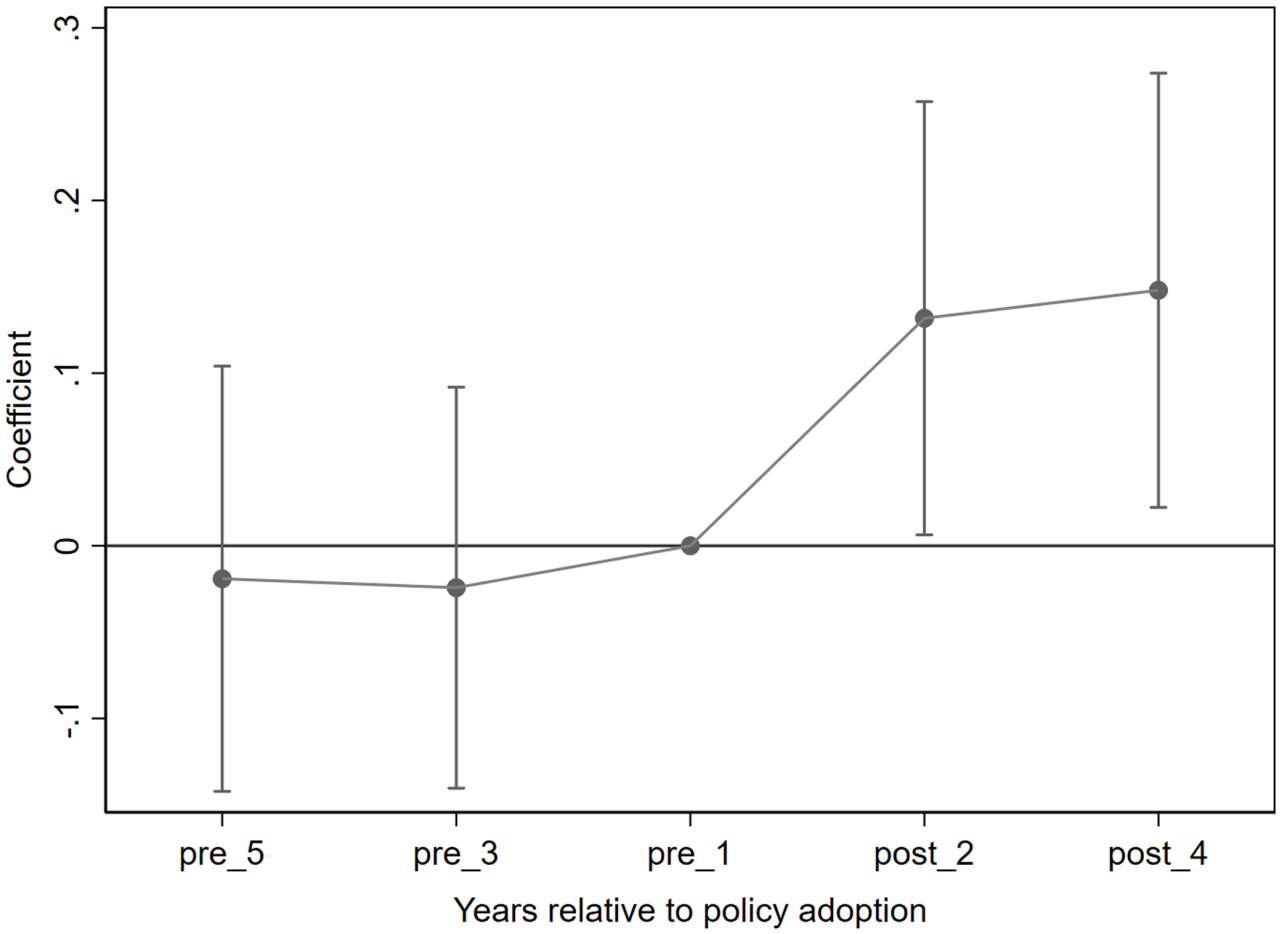
Parallel trend test.

A core assumption of the DID method is that the experimental and control groups exhibit the same trends prior to the policy implementation, known as the parallel trends assumption. To test this assumption, event study analysis was conducted to examine the dynamic changes in income levels of older households before and after the LTCI pilot implementation. The results shown in Figure 2 indicate that prior to the pilot (in 2011, 2013, and 2015), the LTCI pilot variable did not have a significant impact, with the regression coefficients close to zero and confidence intervals including zero. This suggests that the income change trends of the experimental and control groups were consistent before the policy implementation. However, after the pilot (in 2018 and 2020), the DID coefficient was significantly positive, indicating that the LTCI implementation led to an increase in the income levels of older households. This result supports the parallel trends assumption and provides strong evidence for the validity of the baseline regression.

#### 4.2.2 Placebo test

**Figure 3:**
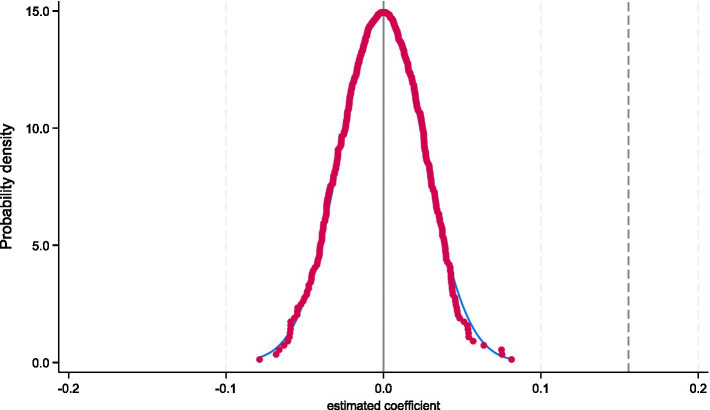
Placebo test.

To further validate the robustness of the policy effect and eliminate the potential interference of unobserved factors, a placebo test was conducted. Specifically, 12 cities were randomly selected from the 126 cities to serve as placebo pilot cities, while the remaining cities were treated as the control group. A regression analysis was conducted on this randomly assigned treatment group to examine whether the randomly set policy pilots had a significant effect on income levels. Figure 3 displays the kernel density distribution of the regression coefficients from 500 random samples, as well as the scatter distribution of *p*-values. The results show that the mean estimated coefficient for the randomly assigned treatment group is close to zero, and most of the p-values are greater than 0.1, indicating no statistical significance. This suggests that the randomly set policy pilots did not have a systematic impact on the income levels of older households, further supporting the robustness of the baseline regression results.

#### 4.2.3 PSM-DID

Due to potential systematic differences in economic development levels, medical resource distribution, and other factors between the pilot and non-pilot regions, which may lead to selection bias, this study employs the PSM-DID method for robustness testing. The propensity score matching method is used to select non-pilot regions with similar socio-economic characteristics to the pilot regions as the control group. Matching methods include nearest-neighbor matching (1:4) and local linear regression matching. After matching, a DID regression is conducted. The results from Models 1 and 2 in Table 3 show that the DID coefficient for the LTCI pilot remains significantly positive, further confirming the policy’s positive impact on the income of older adults.

**Table 3:** Robustness tests: PSM-DID, excluding key provinces, triple difference method, and alternative outcome variables.

Variable	Model 1	Model 2	Model 3	Model 4	Model 5
	PSM-DID		Excluding key provinces	DDD	Replacing dependent variable
did	0.1452^***^	0.1314^*^	0.1096^**^		0.0696^***^
	(0.0471)	(0.0699)	(0.0489)		(0.0213)
DDD				0.1597^***^	
				(0.0479)	
control variables	Yes	Yes	Yes	Yes	Yes
_cons	1.5551^***^	1.7589^***^	1.5060^***^	1.4971^***^	0.9186^***^
	(0.1270)	(0.2182)	(0.1195)	(0.1168)	(0.0519)
time-fixed effects	Yes	Yes	Yes	Yes	Yes
regional-fixed effects	Yes	Yes	Yes	Yes	Yes
*N*	31,604	14,377	29,664	33,435	33,435
adj. *R*^2^	0.3491	0.3692	0.3489	0.3469	0.4093

Standard errors in parentheses.

^*^p < 0.1, ^*^p < 0.05, ^***^p < 0.01.

#### 4.2.4 Excluding key pilot provinces

Considering that Jilin and Shandong provinces are key pilot areas for LTCI in China, they may have unique characteristics in terms of policy implementation, funding investment, and coverage, which could affect the balance of the estimation results. To address this, this study excludes samples from these two provinces and re-conducts the regression analysis. The results from Model 3 in Table 3 show that even after excluding Jilin and Shandong, the DID coefficient for the LTCI pilot remains significantly positive, consistent with the baseline regression results. This suggests that the research conclusions are not influenced by individual key provinces and have strong external validity.

#### 4.2.5 Triple difference (DDD)

To exclude the potential impact of policies other than LTCI on the income of older households and avoid interference with the baseline regression results, this study further constructs a triple difference (DDD) model based on the DID model. Since LTCI only applies to insured individuals, this study groups respondents based on their insurance status to create new “treatment” and “control” groups. Non-insured individuals are unable to benefit from LTCI, and their income changes are primarily influenced by other policies or external factors. Therefore, by comparing the income differences between the original treatment and control groups (which reflect both LTCI and other policy effects) and the new treatment and control groups (which only reflect other policy effects), the net impact of LTCI can be effectively identified. The regression results from Model 4 in Table 3 show that, after controlling for other policy factors, the LTCI pilot still has a significant positive impact on older household income levels, further validating the robustness of the policy effect.

#### 4.2.6 Replacing the dependent variable

To address the right-skewness of the income distribution, the inverse hyperbolic sine (IHS) transformation is applied to the income variable, while avoiding the issues that may arise from handling zero values with a logarithmic transformation. The IHS transformation is a smooth, non-linear transformation that approximates the logarithmic transformation while preserving the original characteristics of the data, improving the robustness of the estimation, and ensuring the comparability of regression results. Additionally, this method balances the variable scale without losing zero-income samples, reducing the influence of extreme values on the regression coefficients and thus enhancing the stability and explanatory power of the model. However, since the coefficient interpretation of the IHS transformation is less intuitive than that of a logarithmic transformation, and its non-linear nature may affect the economic meaning of the baseline regression, this study still uses the raw income values in the baseline regression and introduces the IHS transformation only in the robustness checks to validate the stability of the results. The results from Model 5 in Table 3 show that after replacing the income measure, LTCI still significantly improves older household income, confirming the robustness of the research conclusions.

### 4.3 Mechanism analysis

To explore the specific mechanisms through which LTCI affects older household income, this study introduces employment of older adults and their offspring as potential mediating variables. The employment variable for older adults is based on the survey question, “Did you work for more than one hour last week?” If the response is “yes,” it is considered employment, and the value is assigned as 1; if the response is “no,” the value is assigned as 0. Regarding offspring employment, this study measures the proportion of employed children within a family relative to the total number of children, as this proportion reflects the impact of offspring labor force participation on the family’s economy, thus revealing the potential pathways through which LTCI affects household income.

Table 4, Models 1–2 and Models 3–4, test the mediating role of offspring employment and older adult employment between LTCI policies and older household income. The results indicate that LTCI increases older household income by improving both offspring and older adult employment.

**Table 4:** Mechanism test.

Variable	Model 1	Model 2	Model 3	Model 4
	Employment of children	Income	Employment of older adults	Income
did	0.1471^***^	0.0997^*^	0.0625^***^	0.0731
	(0.0098)	(0.0540)	(0.0196)	(0.0830)
employment of children		0.1573^***^		
		(0.0401)		
employment of older adults				0.3157^***^
				(0.0303)
control variables	Yes	Yes	Yes	Yes
_cons	0.6785^***^	1.4205^***^	0.6315^***^	1.4095^***^
	(0.0192)	(0.1284)	(0.0300)	(0.1398)
time-fixed effects	Yes	Yes	Yes	Yes
regional-fixed effects	Yes	Yes	Yes	Yes
*N*	33,211	29,037	26,439	24,135
adj. *R*^2^	0.0631	0.3487	0.1345	0.3342

Standard errors in parentheses.

^*^p < 0.1, ^**^p < 0.05, ^***^p < 0.01.

The Bootstrap method is used to test the significance of the mediation effect. If the Bootstrap confidence interval for the indirect effect does not include 0, it indicates that the effect is significant. The results in Table 5 show that the indirect effects of offspring employment (95% CI: 0.012, 0.033) and older adult employment (95% CI: 0.002, 0.022) are both significant. This suggests that LTCI can increase older household income by improving both offspring and older adult employment, which is consistent with the previous findings.

**Table 5:** Mediation effect test based on the bootstrap method.

Mediator variable	Effect type	Coefficient	SE	95% conf. interval	
employment of children	Direct effect	0.100	0.064	−0.025	0.225
	Indirect effect	0.022	0.005	0.012	0.033
employment of older adults	Direct effect	0.073	0.079	−0.821	0.228
	Indirect effect	0.012	0.005	0.002	0.022

### 4.4 Heterogeneity analysis

Due to the inherent heterogeneity within the older adult population, the impact of LTCI on household income may vary depending on the characteristics of the group. Table 6 presents the heterogeneous effects of LTCI on older household income. The results indicate that the policy has a significant positive effect on groups with more than two children (Model 2) and on older adults under 75 years old (Model 4). However, the impact is not significant for groups with fewer children (Model 1) or for older adults aged 75 and above (Model 3). This suggests that the income effect of LTCI differs significantly across different older adult groups.

**Table 6:** Heterogeneity analysis.

Variable	Model 1	Model 2	Model 3	Model 4
	Child≤2	Child>2	Age≥75	Age<75
did	0.0554	0.1254^***^	0.0836	0.1701^***^
	(0.1610)	(0.0481)	(0.0808)	(0.0598)
control variables	Yes	Yes	Yes	Yes
_cons	2.2803^***^	1.0269^***^	0.0407	2.5974^***^
	(0.4330)	(0.1169)	(0.2690)	(0.1775)
time-fixed effects	Yes	Yes	Yes	Yes
regional-fixed effects	Yes	Yes	Yes	Yes
*N*	4,092	29,340	10,038	23,397
adj. *R*^2^	0.4011	0.2896	0.4141	0.3318

Standard errors in parentheses.

^*^p < 0.1, ^**^p < 0.05, ^***^p < 0.01.

### 4.5 Further analysis

Table 7 presents the estimated results of LTCI on income inequality among older households, measured using both the Kakwani index and the Yizhaki index. Four models are constructed to ensure the robustness of the results. Models 1 and 3 do not include control variables, while Models 2 and 4 incorporate control variables. All models control for time fixed effects and regional fixed effects. In the regression results using the Kakwani index (Models 1–2), the coefficient of the core explanatory variable DID is −0.0577 (*p* < 0.01) and −0.0622 (*p* < 0.01), respectively. After adding the control variables, the DID coefficient remains statistically significant. In the regression results using the Yizhaki index (Models 3–4), the DID coefficients are −0.0545 (*p* < 0.05) and −0.0432 (*p* < 0.1), also showing a negative impact. Additionally, the inclusion of control variables enhances the explanatory power of the models. The adjusted R^2^ in Models 2 and 4 increased from 0.4129 to 0.4663 and from 0.3422 to 0.3843, respectively, indicating that the inclusion of control variables improves the model’s fit and strengthens the robustness of the estimated results.

**Table 7:** Impact of LTCI on income inequality.

Variable	Model 1	Model 2	Model 3	Model 4
	Kakwani		Yizhaki	
did	−0.0577^***^	−0.0622^***^	−0.0545^**^	−0.0432^*^
	(0.0170)	(0.0196)	(0.0219)	(0.0232)
control variables	No	Yes	No	Yes
_cons	0.7555^***^	0.7050^***^	0.7050^***^	0.6964^***^
	(0.0026)	(0.0320)	(0.0033)	(0.0398)
time-fixed effects	Yes	Yes	Yes	Yes
regional-fixed effects	Yes	Yes	Yes	Yes
*N*	42,163	33,435	42,163	33,435
adj. *R*^2^	0.4129	0.4663	0.3422	0.3843

Standard errors in parentheses.

^*^p < 0.1, ^**^p < 0.05, ^***^p < 0.01.

## 5 Discussion

This study, based on the CHARLS 2011–2020 panel data, employs a DID approach to evaluate the impact of LTCI pilot policies on the income of older households. It further explores the policy’s mechanisms, heterogeneity across groups, and potential effects on income inequality within these households. The findings are as follows:

First, the LTCI pilot policy significantly improved the income levels of older households. The baseline regression analysis indicates that the income-enhancing effect of this policy remains robust across multiple model specifications. This finding is consistent with existing research, which suggests that LTCI improves family economic conditions and enhances quality of life (52, 53). This study further confirms the positive role of social security policies in enhancing individual welfare and extends the understanding of LTCI’s economic impact. The robustness tests show that after controlling for selection bias, and unobserved factors, the results remain consistent, confirming the causal effect of LTCI on older households’ income. Compared to existing studies, this research not only provides a more rigorous causal identification strategy but also expands the analysis of LTCI’s economic consequences, offering more targeted empirical support for policymakers aiming to optimize the social security system. Additionally, it deepens the research framework regarding the impact of social security policies on household economic behavior and income distribution.

Second, the positive impact of LTCI on the income of older households primarily occurs through promoting employment among older individuals and enhancing the employment participation of their offspring. The mechanism analysis shows that LTCI alleviates labor supply restrictions caused by health issues, allowing some labor-capable older individuals to re-enter the labor market or delay their exit, directly increasing household income. Moreover, the policy effectively reduces the pressure on offspring to exit or decrease their labor market participation due to caregiving responsibilities, enabling them to focus more on career development and further increasing overall household income. This result not only extends research on the impact of social security policies on household economic behavior but also deepens the understanding of LTCI’s role in the labor market. The findings align with international studies. For example, Xu and Zweifel (54) and Wang and Liu (55) found that LTCI in China effectively reduced the informal caregiving responsibilities borne by offspring, thereby promoting their participation in the labor market. Coe et al. (56) found that LTCI improved employment levels of offspring in the U.S., and Sugawara and Nakamura (57) confirmed the positive effect of LTCI on female employment in Japan. This study’s mechanism analysis further verified the robustness of the mediating effect through the Bootstrap method, ensuring the reliability of the conclusions. However, unlike existing studies, this research focuses on the context of China’s older adult care system, revealing that LTCI not only enhances offspring employment but also optimizes household resource allocation by encouraging older individuals’ labor participation, thereby boosting overall income. This finding deepens the theoretical framework for understanding the impact of social security policies on the labor market and household economy and provides a new perspective on the economic consequences of LTCI in different institutional environments.

Third, the impact of LTCI on household income among older families demonstrates significant heterogeneity, reflecting the crucial roles of family support and age structure in shaping policy effects. The study finds that the income-enhancing effect of LTCI is more pronounced in households with multiple children (more than 2 children) and among younger older adults (under 75 years old) than in households with fewer children or those with older adults at advanced ages, where the effect is weaker or even insignificant. This heterogeneity highlights the importance of family caregiving resources, labor capacity, and health capital of older adults in the mechanism through which LTCI affects household income. Evidence from European countries indicates that informal caregiving significantly constrains caregivers’ labor market participation, particularly when the duration and intensity of care are substantial (58). From the perspective of family support, the distribution of caregiving responsibilities within families generally follows specific kinship obligations, with distinct divisions of informal care among different family members (59). This suggests that in situations with high caregiving demands, families with multiple members are better able to share caregiving responsibilities, thereby reducing the burden on any single member. This redistribution allows greater labor market participation and enhances overall household income. In contrast, for families with fewer children, especially low- or no-child households, even with partial support from LTCI, substantial residual caregiving needs remain, constraining the employment opportunities of adult children.

From the perspective of age structure, younger older adults (under 75) generally possess stronger labor capacity and health capital. Existing research shows that the formal care support provided by LTCI not only alleviates the informal caregiving burden on family members but also improves the health status of older adults (60), thereby reducing health-related constraints on labor supply and increasing household income. There is a significant negative correlation between the health status of older adults and their labor market participation (61). For older adults aged 75 and above, however, limited health status often restricts their capacity to re-enter the labor force, even with LTCI support. Moreover, this age group typically faces higher medical expenditure pressures (62), which can offset the cost savings from reduced caregiving burdens, thereby weakening the positive income effect of LTCI. These findings align with international evidence. For instance, Fu et al. (63) found that LTCI promotes labor market participation among family members but has limited impact on older adults themselves. This analysis underscores the structural differences in the income effects of LTCI across various family structures. Therefore, policymakers should optimize the delivery model of LTCI to strengthen its economic support for older adults in high-age brackets and for families with fewer children.

Fourth, LTCI plays a positive role in reducing income inequality among older households. Regression analysis shows that, whether using the Kakwani or Yizhaki index, the LTCI pilot policy significantly reduced income disparities within older households. This finding suggests that LTCI, as a social security policy, not only improves the overall income level of the older population but also serves a redistributive function. Existing research has primarily focused on the impact of LTCI on health inequality. For instance, Ke and Sun (64) evaluated the impact of LTCI on health inequality, finding that the policy effectively reduced health disparities within the older population. This study, however, adopts an economic perspective and further reveals LTCI’s unique role in income redistribution in the context of China, enriching research on LTCI’s social effects. Policymakers should further optimize the LTCI supply mechanism to enhance support for low-income older adults. Expanding the coverage of LTCI services for low-income families, increasing subsidies for middle- and low-income groups, and improving the balanced allocation of community caregiving resources will further reduce income disparities within the older population, thereby achieving more inclusive social security goals.

This study contributes to the existing literature in the following ways: First, by employing the CHARLS 2011–2020 panel data and DID method, this research rigorously identifies the causal effect of LTCI pilot policies on older households’ income. Unlike previous studies that mainly focus on LTCI’s impact on health and well-being, this study expands the understanding of LTCI’s economic mechanisms, revealing how it enhances household income by promoting employment among older individuals and their offspring, and ensures the reliability of its conclusions through robustness tests. Second, this study deepens the research on LTCI’s income redistribution effects. While existing studies have focused on LTCI’s impact on health inequality, discussions on income redistribution have been relatively limited. By quantifying the effect of LTCI on income inequality within older households using the Kakwani and Yizhaki indices, this study finds that LTCI not only enhances overall income levels but also effectively reduces income disparities, providing new evidence for the fairness of social security policies. Third, the study reveals the heterogeneous effects of LTCI, expanding the research perspective on social security policies’ impact on household economic behavior. The results indicate that LTCI’s effect on income is more significant in multi-child households and younger older adults, while its impact is weaker in households with fewer children or older adults. This finding goes beyond existing studies that only focus on labor market impacts and highlights the key roles of offspring number and age structure in policy effects, offering empirical support for the precise optimization of LTCI.

While this study provides important findings regarding the economic effects of LTCI, it has the following limitations: First, the heterogeneity of policies is not fully considered. This study did not detail the differences in the implementation intensity, benefit levels, and supporting measures of LTCI pilots in different regions, which may affect the accurate evaluation of policy effects. Future research could incorporate policy text analysis or regional heterogeneity modeling to identify the differentiated impact of LTCI. Second, the long-term effects require further exploration. Due to data limitations, this study mainly focuses on the short- and medium-term effects of LTCI and does not capture its long-term economic effects. Future studies could use data with a longer time span or follow-up surveys to analyze the cumulative impact of the policy on household income, labor market participation, and income distribution. Third, the measurement of the policy’s mechanism is still limited. While this study confirms that LTCI increases household income by promoting employment among older individuals and their offspring, there is a lack of detailed analysis of specific pathways such as employment structure, working hours adjustments, and income optimization. Future research could introduce more refined indicators to comprehensively reveal the policy’s mechanism.

## 6 Conclusion

This study, based on the CHARLS 2011–2020 panel data and employing a DID method, evaluates the impact of LTCI pilot policies on the income of older households. The study finds that LTCI significantly increases household income and reduces income inequality within households by promoting employment among older individuals and their offspring, demonstrating its redistributive potential. Moreover, the policy effects exhibit significant heterogeneity, particularly in multi-child households and younger older adults. The study provides valuable insights for policymakers, suggesting the optimization of LTCI policies based on different family characteristics, such as providing flexible employment support for older adults and reducing the caregiving burden on low- or no-child households. Future research should explore the long-term effects and mechanisms of LTCI, especially its deep impacts on employment types, working hours adjustments, and income structure optimization, to refine policy design and implementation.

## Data Availability

Publicly available datasets were analyzed in this study. This data can be found at: http://charls.pku.edu.cn/en/.
